# Bispecific ANXA2/CD147 CAR-T cell therapy for osteosarcoma

**DOI:** 10.1038/s41388-026-03926-2

**Published:** 2026-08-03

**Authors:** Hai-Jun Tang, Wei Dai, Dan-Ting Xiao, He-Ning Li, Liang Xiong, Ming-Xiu Yang, Ji-Ming Liang, Zhuan Zou, Liang Mai, Shan-Hang Li, Shang-Yu Liu, Wen-Yu Feng, Yun-Hua Lin, Mao-Lin He, Xin-Li Zhan, Yun Liu

**Affiliations:** 1 Department of Spine and Osteopathic Surgery, The First Affiliated Hospital of Guangxi Medical University, Nanning, Guangxi China; 2 Department of Oncology, The First Affiliated Hospital of Guangxi Medical University, Nanning, Guangxi China; 3 Department of Bone and Soft Tissue Tumor, Tumor Hospital of Guangxi Medical University, Nanning, Guangxi China; 4 Department of Bone and Joint Surgery and Sports Medicine, The Second Affiliated Hospital of Guangxi Medical University, Nanning, China

**Keywords:** Bone cancer, Immunotherapy

## Abstract

Osteosarcoma is a highly malignant tumor with poor prognosis. Current CAR-T cell therapies for osteosarcoma are predominantly designed with single targets, but their efficacy remains unsatisfactory. In this study, a novel bispecific CAR-T cell was developed to provide an experimental basis for improving the therapeutic outcome of osteosarcoma. Single-cell RNA sequencing (scRNA-seq) identified two antigens highly expressed in osteosarcoma cells, ANXA2 and CD147, whose expression was further validated at the tissue level by qRT-PCR, flow cytometry, and immunohistochemistry. Based on a second-generation CAR backbone, a bispecific ANXA2/CD147 CAR-T construct was generated using magnetic bead sorting, primary T-cell culture, and lentiviral transduction, achieving a transduction efficiency of 47.1%. LDH release assays demonstrated that bispecific CAR-T cells exhibited significantly greater cytotoxicity against tumor cells than single-target and control groups. ELISA confirmed that bispecific CAR-T cells released higher levels of effector molecules, including GZMB and TNFα. In a subcutaneous CDX model, bispecific CAR-T cells displayed superior antitumor activity and greater T-cell infiltration. In a paw pad xenograft model, mice treated with bispecific CAR-T cells exhibited the smallest tumor volumes, lowest tumor weights, and reduced rates of lymph node metastasis. Furthermore, PDX models confirmed that bispecific CAR-T cells effectively suppressed osteosarcoma growth. ScRNA-seq of tumors derived from CDX models and immunohistochemistry revealed markedly increased infiltration of M1 macrophages in the bispecific group. Collectively, this study successfully generated a bispecific ANXA2/CD147 CAR-T cell with robust antitumor activity, providing a promising new strategy for the immunotherapy of osteosarcoma.

## Introduction

Osteosarcoma (OS) is the most common malignant bone tumor, primarily affecting the long bones of adolescents. It is characterized by high aggressiveness and an early propensity for distant metastasis, posing a serious threat to both the physical and mental health of young patients [1]. The current standard of care—comprising neoadjuvant chemotherapy, surgical resection, and adjuvant chemotherapy—has markedly improved the 5-year survival rate of patients. However, prognosis remains unsatisfactory: the 5-year survival rate is approximately 60% for localized disease, but falls to below 30% for patients with metastases [2, 3]. Despite the approval of several novel anticancer agents in recent years, overall survival has not improved significantly [4]. Thus, the development of innovative therapeutic strategies is of critical importance for improving patient outcomes.

Adoptive cell therapy (ACT) involves the collection of autologous immune cells, followed by genetic engineering and ex vivo expansion, after which the modified cells are reinfused into the patient to mediate antitumor activity [5]. Among ACT approaches, chimeric antigen receptor T (CAR-T) cell therapy represents the most advanced and widely applied modality [6]. Multiple CAR-T products have already achieved clinical translation and demonstrated remarkable efficacy in hematological malignancies [7, 8]. Although several preclinical studies have demonstrated the therapeutic potential of CAR-T cells in osteosarcoma [9, 10], the results of clinical studies have been unsatisfactory. In 2015, the results of a phase I/II clinical trial (NCT00902044) involving 19 HER2-positive sarcoma patients (including 16 with osteosarcoma) showed that escalating doses of HER2-CAR-T cells (1 × 10^4^ to 1 × 10^8^/m^2^) were well tolerated. Nonetheless, among 14 evaluable osteosarcoma patients, none achieved remission, four achieved stable disease, and the remainder experienced progression [11]. More recently, in 2024, a phase I prospective study enrolled 14 patients with refractory sarcomas who received HER2-CAR-T infusion at 1 × 10^8^/m^2^. Of the eight osteosarcoma patients included, only one achieved complete remission, two achieved stable disease, and the remainder progressed [12]. These findings underscore the urgent need for the development and optimization of CAR-T therapies to improve survival in osteosarcoma patients.

Osteosarcoma is a solid tumor with profound intratumoral heterogeneity, characterized by the coexistence of multiple tumor cell subpopulations within the same lesion, each expressing distinct tumor antigens [13, 14]. This heterogeneity markedly reduces the recognition and cytotoxic activity of CAR-T cells. To address this challenge, bispecific CAR-T cells have been designed, in which two different single-chain variable fragments (scFvs) are linked within a single CAR construct. This design enables CAR-T cells to be activated by recognizing either antigen, thereby enhancing their capacity to initiate antitumor responses. Compared with conventional single-target CAR-T cells, bispecific CAR-T cells have demonstrated superior efficacy in multiple solid tumors [15–17]. However, reports of their application in osteosarcoma remain scarce.

In the present study, we identified and validated the high expression of ANXA2 and CD147 in osteosarcoma and constructed a novel bispecific ANXA2/CD147 CAR-T cell using lentiviral transduction of primary T cells. Its antitumor efficacy was confirmed in vitro and in multiple in vivo models. Furthermore, single-cell RNA sequencing (scRNA-seq) revealed that treatment with bispecific CAR-T cells promoted enhanced infiltration of M1 macrophages within the tumor microenvironment. To the best of our knowledge, this is the first report of bispecific ANXA2/CD147 CAR-T cells applied in osteosarcoma therapy.

## Materials and methods

### scRNA-seq of human osteosarcoma tissues and data processing

To identify tumor antigens specifically overexpressed in osteosarcoma cells, scRNA-seq was performed on clinical samples from 14 osteosarcoma patients, including primary tumors (PT, *n* = 14), adjacent normal tissues (ANT, *n* = 6), metastatic lymph nodes (MLN, *n* = 2), and negative lymph nodes (NLN, *n* = 4). In addition, publicly available osteosarcoma datasets from the GEO database (GSE152048, *n* = 7) and femoral head samples (GSE169396, *n* = 4) were collected for validation of target expression.

Following enzymatic digestion, single-cell suspensions were prepared and sequenced using the 10 × Genomics platform. Raw sequencing reads were subjected to quality control and processed with Cell Ranger (v7.1.0) using GRCh38 as the reference genome. Data were converted into Seurat objects (Seurat v4.0.5). Cells with 500–5000 detected genes and <15% mitochondrial gene content were retained. Normalization was performed using the SCTransform function, and batch effects were corrected using the Harmony algorithm. Dimensionality reduction was conducted with principal component analysis (PCA, top 15 PCs), followed by clustering with uniform manifold approximation and projection (UMAP). Differentially expressed genes (DEGs) across clusters were identified with the FindAllMarkers function, and cell types were annotated according to canonical markers and published references. Group-specific DEGs were determined using the FindMarkers function.

### scRNA-seq of mouse tumor tissues and data processing

To investigate the impact of CAR-T therapy on the tumor microenvironment, tumors from 12 mice were collected for scRNA-seq. Single-cell suspensions were sequenced on the DNBSEQ-T7 platform. Quantification was performed with Cell Ranger (v7.1.0). Cells with 500–4500 detected genes and <25% mitochondrial content were retained. Cell origin (human osteosarcoma cell line–derived vs. mouse-derived) was identified based on the cell_classification.csv file. Data were normalized with NormalizeData (scale.factor = 10,000), and 2000 variable features were identified with FindVariableFeatures. Dimensionality reduction was performed using PCA (top 15 PCs), followed by clustering with FindClusters and visualization with RunUMAP.

### GSVA analysis

Gene set variation analysis (GSVA) was performed using murine GO gene sets obtained from the msigdbr package (v7.5.1). Enrichment scores for M1- and M2-associated gene sets were calculated. Differential pathway analysis was conducted using the limma package (v3.50.3), with thresholds set at logFC > 0.5 and adjusted *p* < 0.05.

### MiloR analysis

The MiloR algorithm was applied to assess differential cell infiltration across treatment groups. Seurat objects were subset by group and converted into SingleCellExperiment objects. A k-nearest neighbor (k-NN) graph was constructed (k = 30, d = 50), and neighborhoods were generated with random sampling (prop = 0.2, refined = TRUE). Cell counts were aggregated by sample identity, and neighborhood distances were computed (d = 50). Differential abundance testing was performed using testNhoods (~group_trend), and results were annotated by cell type.

### Quantitative reverse transcription PCR (qRT-PCR)

Fresh osteosarcoma and adjacent normal tissues were homogenized using a motorized homogenizer. Total RNA was extracted from tissues and cell lines with the Hipure Total RNA Mini Kit (Magen, China). cDNA was synthesized with a reverse transcription kit (Takara, Japan) in a 20 μL reaction volume. qRT-PCR was performed using the FastStart Universal SYBR Green Master ROX kit (Roche, Germany) on a QuantStudio™ platform. Relative expression levels of target genes were calculated using the 2^−ΔΔCT^ method. Primer sequences for ANXA2, CD147, and GAPDH are provided in Supplementary Table S1.

### Flow cytometry

Fresh surgical samples were mechanically dissociated into a minced state and filtered through a 70 μm strainer. After 24 h culture, adherent cells were collected. Osteosarcoma cell lines and primary T cells were prepared as single-cell suspensions for analysis. Cells were incubated with the following antibodies for 30 min at room temperature in the dark: FITC anti-human CD3 (BioLegend, Cat#317305, Clone OKT3, 5 μL/10⁶ cells); PE anti-human CD4 (BioLegend, Cat#317409, Clone OKT4, 5 μL/10⁶ cells); APC anti-human CD8 (BioLegend, Cat#344721, Clone SK1, 5 μL/10⁶ cells); PE anti-human CD147 (BioLegend, Cat#306211, Clone HIM6, 5 μL/10⁶ cells); and anti-Annexin A2/ANXA2 (Abcam, Cat#ab178677, Clone EPR13052(B), 1:50 dilution).

### Immunohistochemistry (IHC)

Osteosarcoma and metastatic lymph node tissues were fixed in 4% paraformaldehyde (PFA), washed, dehydrated in graded ethanol, cleared in xylene, and embedded in paraffin. Sections were cut, deparaffinized, and rehydrated. Antigen retrieval was performed with EDTA buffer. Endogenous peroxidase activity was blocked with 3% hydrogen peroxide for 15 min, followed by blocking with goat serum for 15 min. Sections were incubated overnight at 4 °C with primary antibodies against ANXA2 (Abcam, UK, 1:50) or CD147 (Abcam, UK, 1:3000), followed by HRP-conjugated secondary antibodies. DAB chromogen was applied for 5–10 min, and nuclei were counterstained with hematoxylin. After dehydration and clearing, slides were mounted with neutral resin. Femoral head tissue sections were obtained from patients with femoral neck fractures (courtesy of the pathology department).

### Plasmid construction and lentiviral packaging

The scFv sequence for CD147 was obtained from a published report [18], and the scFv for ANXA2 was derived from patent EP4356972. The bispecific CAR construct was designed by linking the two scFvs with a 3 × EAAK linker. All CAR constructs contained 4-1BB as the intracellular co-stimulatory domain and CD3ζ as the signaling domain. Plasmids were synthesized by Cyagen (Suzhou, China). 293T cells were transfected with plasmids using the GeneReal So-easy kit (Presci BioTech, Cat#L002C-20T), and lentiviral particles were harvested and concentrated using the Presci lentivirus concentration kit (Cat#L020PS50).

### Generation of CAR-T cells

Peripheral blood lymphocytes were isolated from healthy donors using Ficoll-Paque (Cytiva, USA). CD3⁺ T cells were enriched with a positive selection kit (STEMCELL Technologies, Canada, Cat#17851) and stimulated with CD3/CD28 activators (STEMCELL, Cat#10971). When T cells entered proliferative clusters, they were transduced with lentiviral particles in the presence of polybrene (5 μg/mL) by centrifugation (32 °C, 1000 × *g*, 120 min). T cells were cultured and expanded in ImmunoCult™-XF medium (STEMCELL, Cat#10981). Transduction efficiency and T-cell phenotypes were evaluated by flow cytometry.

### Cell culture

Osteosarcoma cell lines 143B, SAOS2, and MG63 were obtained from the Cell Bank of the Chinese Academy of Sciences. The hFOB 1.19 cell line was purchased from Cyagen, and HEK293T cells from Jinyuan Biotechnology (Shanghai, China). 143B, MG63, and 293T cells were cultured in DMEM (Gibco, USA) supplemented with 10% FBS and 1% penicillin/streptomycin. SAOS2 cells were cultured in McCoy’s 5A medium with 15% FBS and 1% penicillin/streptomycin. hFOB 1.19 cells were maintained in DMEM/F-12 medium supplemented with 10% FBS and 1% penicillin/streptomycin. Primary tumor cells and primary T cells were cultured in RPMI-1640 supplemented with 10% FBS and 1% penicillin/streptomycin.

### Cytotoxicity assay

CAR-T cells were co-cultured with osteosarcoma cell lines (143B, SAOS2, MG63) at effector-to-target (E:T) ratios of 1:1, 10:1, and 20:1 for 24 h. Supernatants were collected, and cytotoxicity was quantified using a Cytotoxicity LDH Assay Kit (MCE, Cat#HY-K1090).

### Cytokine production

CAR-T cells were co-cultured with osteosarcoma cell lines at an E:T ratio of 10:1 for 24 h. Supernatants were analyzed for GZMB and TNFα using ELISA kits (Human GZMB ELISA, DL-GZMB-Hu; Human TNFα ELISA, DL-TNFa-Hu; R&D Systems) according to the manufacturer’s instructions. Absorbance was measured at 450 nm using a Tecan microplate reader.

### Crystal violet staining

Crystal violet staining was performed to assess CAR-T-cell-mediated cytotoxicity against osteosarcoma cells. Briefly, 143B and MG63 cells, representing osteosarcoma cell lines with high and low expression of ANXA2 and CD147, respectively, were seeded into 24-well plates at a density of ~5 × 10⁴ cells per well. When the tumor cells reached 60–70% confluence, they were co-cultured with MOCK-CAR-T, ANXA2-CAR-T, CD147-CAR-T, or ANXA2-CD147-CAR-T cells for 48 h. After co-culture, non-adherent cells were removed, and the remaining adherent tumor cells were washed two to three times with PBS and fixed with 4% paraformaldehyde for 15 min at room temperature. The fixed cells were then washed with PBS and stained with 0.1% crystal violet solution for 10–20 min. Excess dye was gently removed by washing with PBS, and the plates were air-dried before imaging under an upright microscope.

### Establishment of subcutaneous CDX models

SPF-grade female BALB/c-nu mice aged 4 weeks were used for the in vivo experiments. After a 7-day acclimatization period, 143B cells in the logarithmic growth phase were harvested, washed with PBS, and resuspended to the required concentration. To establish the subcutaneous xenograft model, 2 × 10⁶ 143B cells suspended in 100 μL of sterile PBS were subcutaneously injected into the right dorsal flank of each mouse. To establish the paw-pad lymph node metastasis model, 2 × 10⁶ 143B cells suspended in 50 μL of sterile PBS were subcutaneously injected into the paw pad of each mouse. After tumor formation was observed, the mice were randomly assigned to different treatment groups, with five mice in each group. Tumor formation, body weight, and general health status were monitored regularly.

### Establishment of PDX models

PDX models were generated in 4-week-old female NCG mice (15–18 g). A 4–6 mm skin incision was made parallel to the spine, and subcutaneous and fascial tissues were dissected. Fresh osteosarcoma specimens from patients were cut into 3 × 3 × 3 mm³ fragments and implanted subcutaneously. When the first-generation tumors reached ~1000 mm³, they were excised, sectioned into equal fragments, and re-implanted into 4-week-old BALB/c-nu female mice for expansion. After tumor formation was observed, the mice were randomly assigned to different treatment groups, with five mice in each group.

### Treatment administration

CAR-T cells were administered intravenously (1 × 10⁶ cells per mouse, 50 μL per injection) twice on days 0 and 5 after tumor establishment. Cisplatin was administered intraperitoneally at 2 mg/kg once every 7 days for two doses, based on human-to-mouse equivalent dosing.

### H&E staining of mouse liver tissues

After treatment, liver tissues from the left lobe were collected from PDX model mice in each group and fixed in 4% paraformaldehyde for 24 h. The tissues were then routinely dehydrated through a graded ethanol series, cleared in xylene, embedded in paraffin, and sectioned at a thickness of 4 μm. After deparaffinization and rehydration, the sections were stained with hematoxylin for 5–10 min, differentiated in acid alcohol, blued, and rinsed under running water. The sections were then stained with eosin for 1–3 min, dehydrated, cleared, and mounted with neutral resin. Pathological changes in liver tissues were observed and imaged under a light microscope.

### siRNA transfection

Three candidate siRNA sequences targeting human CD147 were designed, including si-CD147-717, si-CD147-460, and si-CD147-891, and synthesized by Wuhan GeneCreate Biological Engineering Co., Ltd. The detailed sequences are listed in Supplementary Table S2. siRNA transfection into 143B cells was performed using the Sangon™ RNA TransMate transfection reagent kit (Cat. No. E607402) according to the manufacturer’s instructions. After transfection, the mRNA expression level of CD147 was detected by qRT-PCR, and the siRNA sequence with the highest knockdown efficiency, si-CD147-891, was selected for subsequent experiments.

### qRT-PCR analysis of macrophage polarization

THP-1 cells were treated with 100 ng/mL phorbol 12-myristate 13-acetate (PMA) for 48 h to induce adherent M0 macrophage-like cells. CD147-silenced 143B cells, referred to as the KD group, and untreated wild-type 143B cells, referred to as the WT group, were separately co-cultured with M0 macrophages in an co-culture system. After 48 h of co-culture, macrophages were collected, and total RNA was extracted. The expression levels of M1 macrophage polarization-related genes, including TNF-α and CD86, and M2 macrophage polarization-related genes, including MRC1 and CD163, were examined by qRT-PCR. The primer sequences of the genes are listed in Supplementary Table S1.

### IHC analysis of M1 and M2 macrophage markers in PDX tumors

Tumor tissues from PDX model mice in each treatment group were fixed, embedded, and sectioned. After antigen retrieval and blocking, the sections were incubated with primary antibodies against CD206 at 1:100, iNOS at 1:150, and CD86 at 1:100, followed by incubation with an HRP-conjugated secondary antibody at 1:200. DAB staining was then performed, followed by hematoxylin counterstaining. Images were observed and captured using a research-grade upright microscope. Finally, the positively stained area was quantified using ImageJ, and statistical analysis and graph generation were performed using GraphPad Prism.

### Immunofluorescence

Tumor tissues were fixed in PFA for 4 h, dehydrated in 30% sucrose, embedded, and frozen at −80 °C. Sections were permeabilized with 0.3% Triton X-100 for 20 min, blocked with serum for 30 min, and incubated overnight at 4 °C with primary anti-CD3 antibody (BioLegend, 1:400). After washing, sections were incubated with secondary antibody (BioLegend, 1:400) for 75 min at room temperature, counterstained with DAPI for 30 min, and mounted. Fluorescence signals were observed with a confocal microscope.

### Statistical methods

No formal sample size calculation was performed. Sample sizes were determined based on previous studies and preliminary experiments using similar osteosarcoma CDX and PDX models. No samples or animals were excluded from the analyses. For animal experiments, the exact number of mice in each group is indicated in the corresponding figure legends. Blinding was not performed during treatment administration or outcome assessment.

Bioinformatic analyses (e.g., single-cell sequencing data processing) were performed using R software (version 4.1.0). Differential analyses between groups were conducted using the Wilcoxon rank-sum test. Cell-based and animal experimental data are presented as mean ± standard deviation (SD). Comparisons between two groups were performed using independent-samples *t*-tests, while comparisons among multiple groups were assessed using one-way analysis of variance (ANOVA). All statistical analyses were conducted with SPSS software (version 22.0). A *P*-value < 0.05 was considered statistically significant. Significance levels were defined as follows: *P < 0.05; **P < 0.01; ***P < 0.001; ****P < 0.0001.

## Results

### ANXA2 and CD147 are highly expressed in osteosarcoma and metastatic lymph nodes

**Fig. 1:**
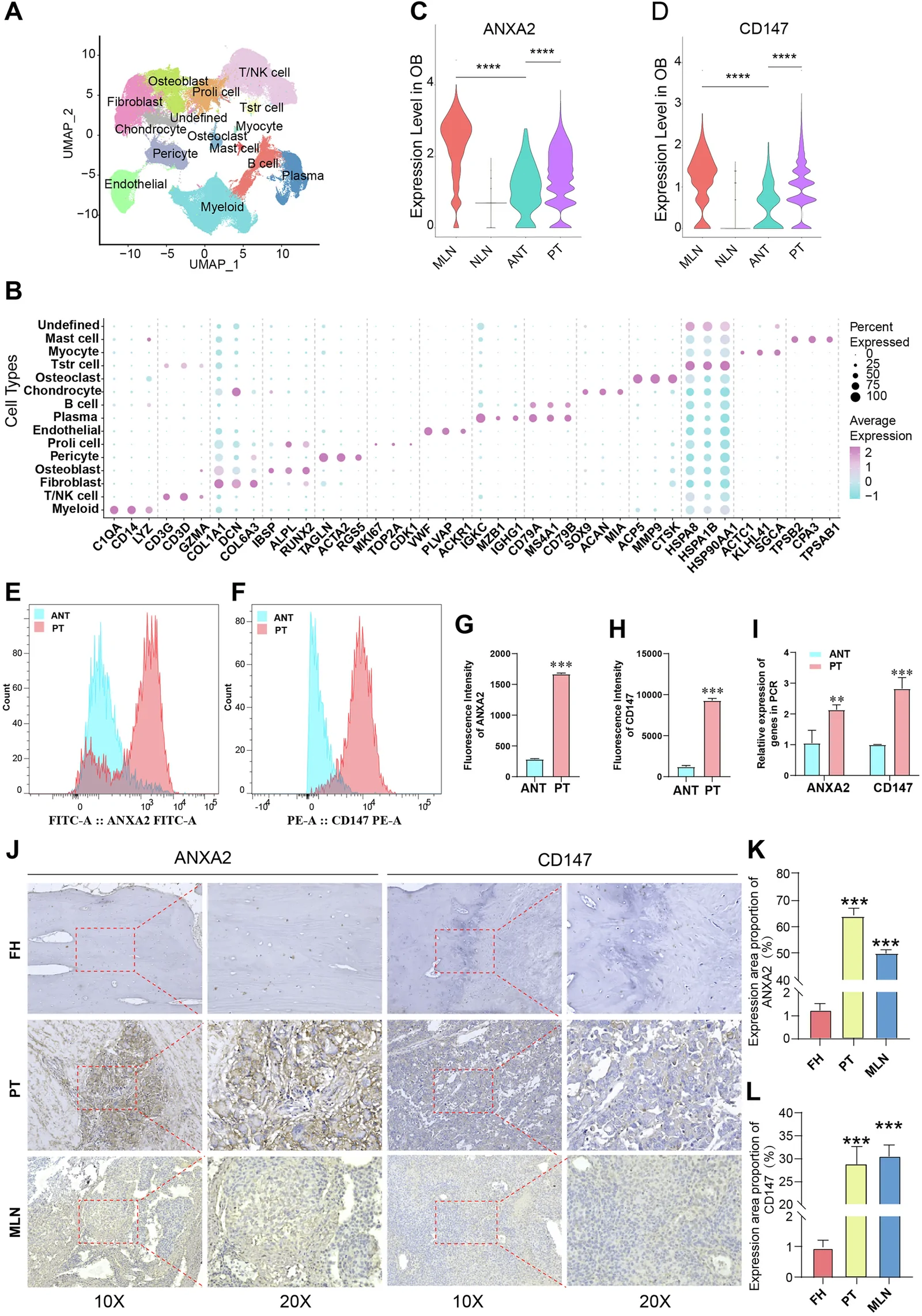
ANXA2 and CD147 are highly expressed in osteosarcoma and metastatic lymph nodes. **A** UMAP visualization of scRNA-seq data from human osteosarcoma (PT, *n* = 14), adjacent normal tissue (ANT, *n* = 6), metastatic lymph nodes (MLN, *n* = 2), and negative lymph nodes (NLN, *n* = 4). A total of 233,087 cells were clustered into 15 subpopulations. **B** Expression of canonical marker genes used to annotate each cell cluster. **C**, **D** Violin plots showing ANXA2 and CD147 expression levels, which were significantly elevated in MLN and PT compared with ANT, and nearly absent in NLN. **E**–**H** Flow cytometry analysis of ANXA2 and CD147 expression in osteosarcoma tissues versus adjacent normal tissues (*n* = 3). **I** qRT-PCR further confirmed increased transcript levels of ANXA2 and CD147 in osteosarcoma tissues compared with adjacent tissues. **J** Representative immunohistochemistry (IHC) images of ANXA2 and CD147 expression in femoral head (FH), PT, and MLN samples (*n* = 3). **K**–**L** Quantification of ANXA2 and CD147 expression area in FH, PT, and MLN tissues. Statistical analysis: Wilcoxon rank-sum test was used for (**C**) and (**D**). Student’s *t* test was used for (**G**–**I**, **K**, **L**). All tests were two-sided. *P < 0.05, **P < 0.01, ***P < 0.001, ns not significant.

To identify tumor-associated antigens that highly expressed in osteosarcoma, single-cell RNA sequencing (scRNA-seq) was performed on 26 clinical samples, including primary tumors (PT, *n* = 14), adjacent normal tissues (ANT, *n* = 6), metastatic lymph nodes (MLN, *n* = 2), and negative lymph nodes (NLN, *n* = 4). After quality control, 233,087 cells were retained for dimensionality reduction and clustering, yielding 15 distinct clusters (Fig. 1A). Each cluster was annotated based on canonical marker gene expression (Fig. 1B). Cluster 4 displayed high expression of IBSP, ALPL, and RUNX2, which are associated with osteoblast differentiation, and was therefore annotated as osteoblasts (OB). Previous studies have shown that, compared with OBs from adjacent tissues, OBs in osteosarcoma exhibit greater copy number variations, higher malignancy, and enriched expression of tumor-promoting genes; thus, these OBs were classified as osteosarcoma (OS) cells [19].

Differential expression analysis between OS cells and OBs revealed that ANXA2 (Fig. 1C) and CD147 (Fig. 1D) were significantly upregulated in osteosarcoma tissues and metastatic lymph nodes. Consistent results were observed in published osteosarcoma scRNA-seq datasets, where ANXA2 and CD147 expression remained markedly higher in OS cells compared with OBs (Supplementary Fig. S1A–C).

To validate these findings, flow cytometry demonstrated significantly higher ANXA2 and CD147 expression in osteosarcoma tissues compared with adjacent normal tissues (Fig. 1E–H). qRT-PCR further confirmed elevated transcript levels of ANXA2 and CD147 in osteosarcoma (Fig. 1I), and immunohistochemistry verified robust protein expression of both markers (Fig. 1J–L). Both ANXA2 and CD147 encode membrane-associated proteins and are suitable as tumor antigens [20, 21]. Based on these results, ANXA2 and CD147 were selected as target antigens for CAR-T cell construction.

### Construction and characteristics of ANXA2-CD147-CAR-T cells

**Fig. 2:**
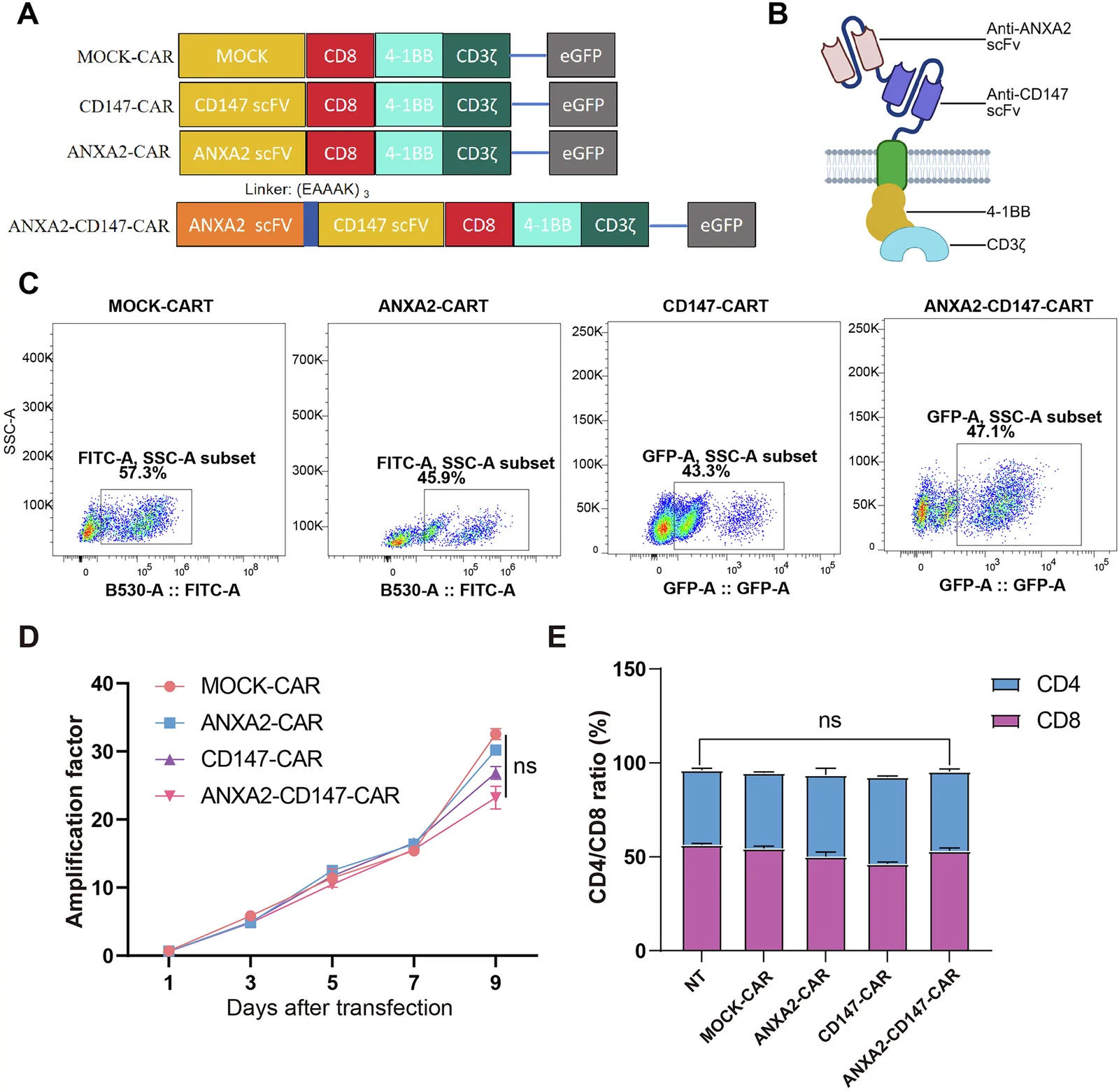
Construction and characteristics of ANXA2-CD147-CAR-T cells. **A**, **B** Schematic diagrams of CAR constructs. The bispecific ANXA2-CD147 CAR was generated by tandemly linking ANXA2- and CD147-specific scFvs with a rigid EAAK linker. MOCK-CAR lacking antigen recognition and two monospecific CARs (ANXA2-CAR and CD147-CAR) were designed as controls. **C** Flow cytometry analysis of transduction efficiency. Transduction rates were 57.3% for MOCK-CAR-T, 45.9% for ANXA2-CAR-T, 43.3% for CD147-CAR-T, and 47.1% for ANXA2-CD147-CAR-T cells. **D** Growth kinetics of CAR-T cells showing comparable expansion among groups, with all reaching ~30-fold amplification by day 9 post-transduction. **E** Flow cytometry analysis of CAR-T cell subsets showing no significant differences in CD4⁺/CD8⁺ ratios between CAR-T groups and non-transduced T cells (NT). Statistical analysis: comparisons in (**E**) were performed using Student’s *t* test. ns not significant.

A second-generation ANXA2-CD147 CAR construct (hereafter referred to as bispecific-CAR) was generated, in which ANXA2-specific and CD147-specific single-chain variable fragments (scFvs) were linked in tandem via a rigid EAAK linker. For comparison, a mock CAR lacking antigen recognition (MOCK-CAR) and two monospecific CAR constructs (ANXA2-CAR and CD147-CAR) were also designed (Fig. 2A, B).

Peripheral blood mononuclear cells (PBMCs) from healthy donors were isolated, and CD3⁺ T cells were enriched by magnetic bead sorting. Flow cytometry confirmed a purity of 93.4% for CD3⁺ T cells (Supplementary Fig. S2A). Transduction efficiency was subsequently evaluated by flow cytometry, showing rates of 57.3% for MOCK-CAR-T cells, 45.9% for ANXA2-CAR-T cells, 43.3% for CD147-CAR-T cells, and 47.1% for bispecific-CAR-T cells (Fig. 2C). Representative images of bispecific-CAR–transduced T cells are shown in Supplementary Fig. S2B.

Growth kinetics analysis demonstrated that all CAR-T groups expanded efficiently, reaching approximately 30-fold expansion by day 9 post-transduction, indicating that incorporation of the bispecific CAR did not impair T-cell proliferation (Fig. 2D). Flow cytometry revealed no significant differences in the proportions of CD4⁺ and CD8⁺ T cells among CAR-T groups compared with non-transduced T cells (NT) (Fig. 2E).

### ANXA2-CD147-CAR-T cells efficiently kill OS cells in vitro

To select osteosarcoma cell lines with differential antigen expression, ANXA2 and CD147 levels were assessed in 143B, SAOS2, and MG63 cells by flow cytometry. CD147 and ANXA2 were most highly expressed in 143B, moderately expressed in SAOS2, and lowest in MG63 (Supplementary Fig. S3A–C). qRT-PCR confirmed the same expression gradient (Supplementary Fig. S3D–E). Thus, these three cell lines provided an antigen-gradient system to evaluate CAR-T cytotoxicity.

**Fig. 3:**
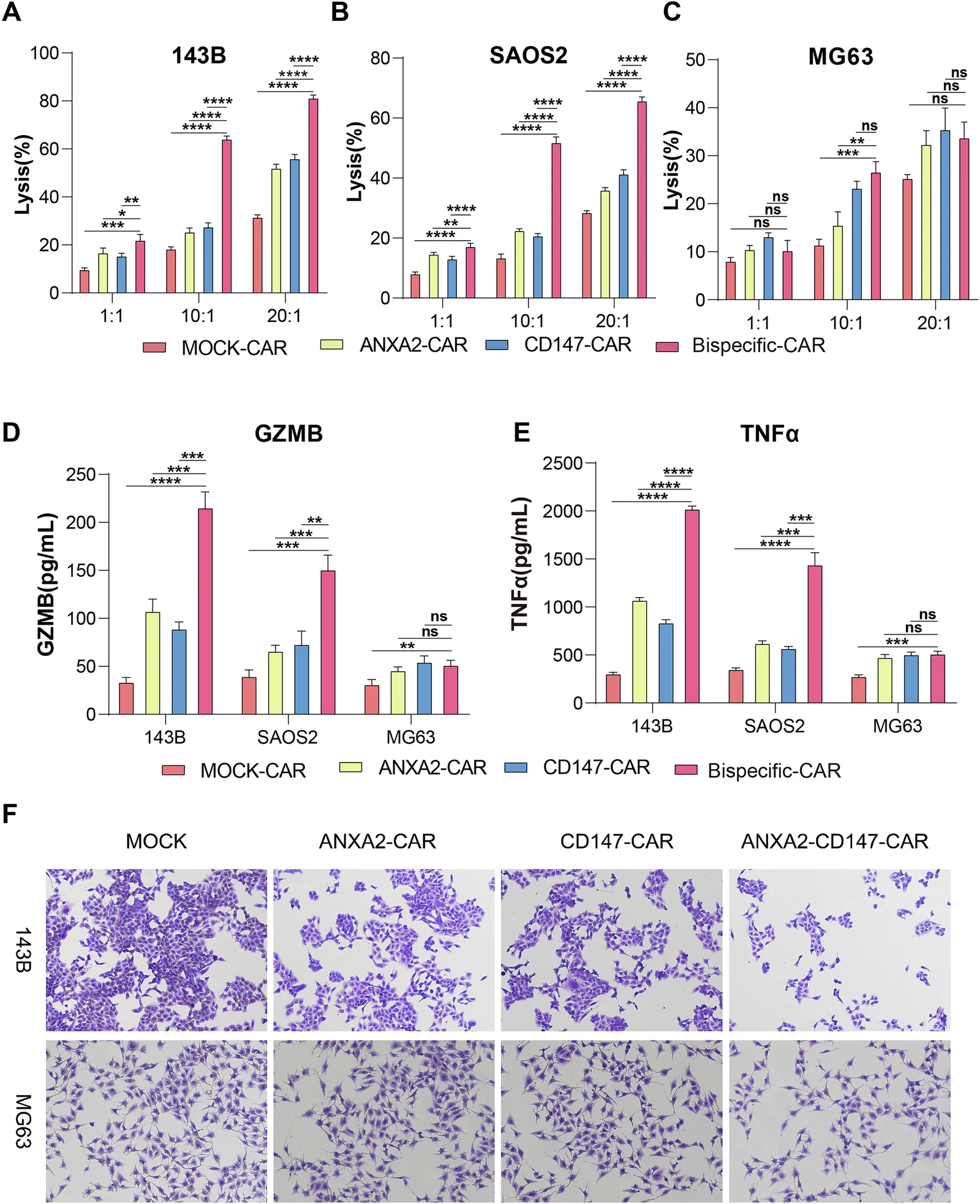
ANXA2-CD147-CAR-T cells efficiently kill osteosarcoma cells in vitro. **A**–**C** LDH release assays evaluating cytotoxicity of MOCK-CAR-T, ANXA2-CAR-T, CD147-CAR-T, and ANXA2-CD147-CAR-T cells against osteosarcoma cell lines 143B, SAOS2, and MG63 at effector-to-target (E:T) ratios of 1:1, 10:1, and 20:1. (*n* = 3). **D**, **E** ELISA quantification of GZMB and TNFα secretion by CAR-T cells co-cultured with 143B, SAOS2, and MG63 at an E:T ratio of 10:1 (*n* = 3). **F** Crystal violet staining of residual adherent 143B and MG63 cells after co-culture with different CAR-T cells (*n* = 3). Statistical analysis: Student’s *t* test was used for all comparisons. All tests were two-sided. *P < 0.05, **P < 0.01, ***P < 0.001, ****P < 0.0001, ns not significant.

LDH release assays showed that at effector-to-target (E:T) ratios of 1:1, 10:1, and 20:1, bispecific-CAR-T cells exhibited the highest cytotoxicity against 143B cells (double-positive for ANXA2 and CD147), significantly exceeding monospecific and MOCK groups (Fig. 3A). Against SAOS2 cells, cytotoxicity of bispecific-CAR-T cells remained higher than that of monospecific and MOCK groups, although overall levels were lower than in 143B (Fig. 3B). Against MG63 cells, which expressed low levels of both antigens, cytotoxicity was largely comparable among groups (Fig. 3C).

At an E:T ratio of 10:1, ELISA results showed that bispecific-CAR-T cells secreted significantly higher levels of GZMB and TNFα when co-cultured with 143B cells, compared with monospecific and MOCK group. With SAOS2, cytokine secretion was reduced relative to 143B, whereas cytokine secretion in MG63 co-cultures was largely comparable among groups (Fig. 3D, E).

Furthermore, we performed crystal violet staining to assess tumor cell death following co-culture with CAR-T cells. The results showed that, when co-cultured with 143B cells, which are double-positive for the target antigens, the bispecific CAR-T group exhibited markedly fewer residual adherent tumor cells than the monospecific CAR-T and control groups, indicating enhanced tumor cell killing. In contrast, when co-cultured with MG63 cells, no obvious differences in the number of adherent tumor cells were observed among the groups (Fig. 3F).

Taken together, these findings indicate that bispecific CAR-T cells can effectively target tumor cells expressing ANXA2 and/or CD147, leading to stronger cytotoxicity and cytokine secretion than monospecific CAR-T cells.

### ANXA2-CD147-CAR-T cells suppress osteosarcoma growth in subcutaneous CDX models

**Fig. 4:**
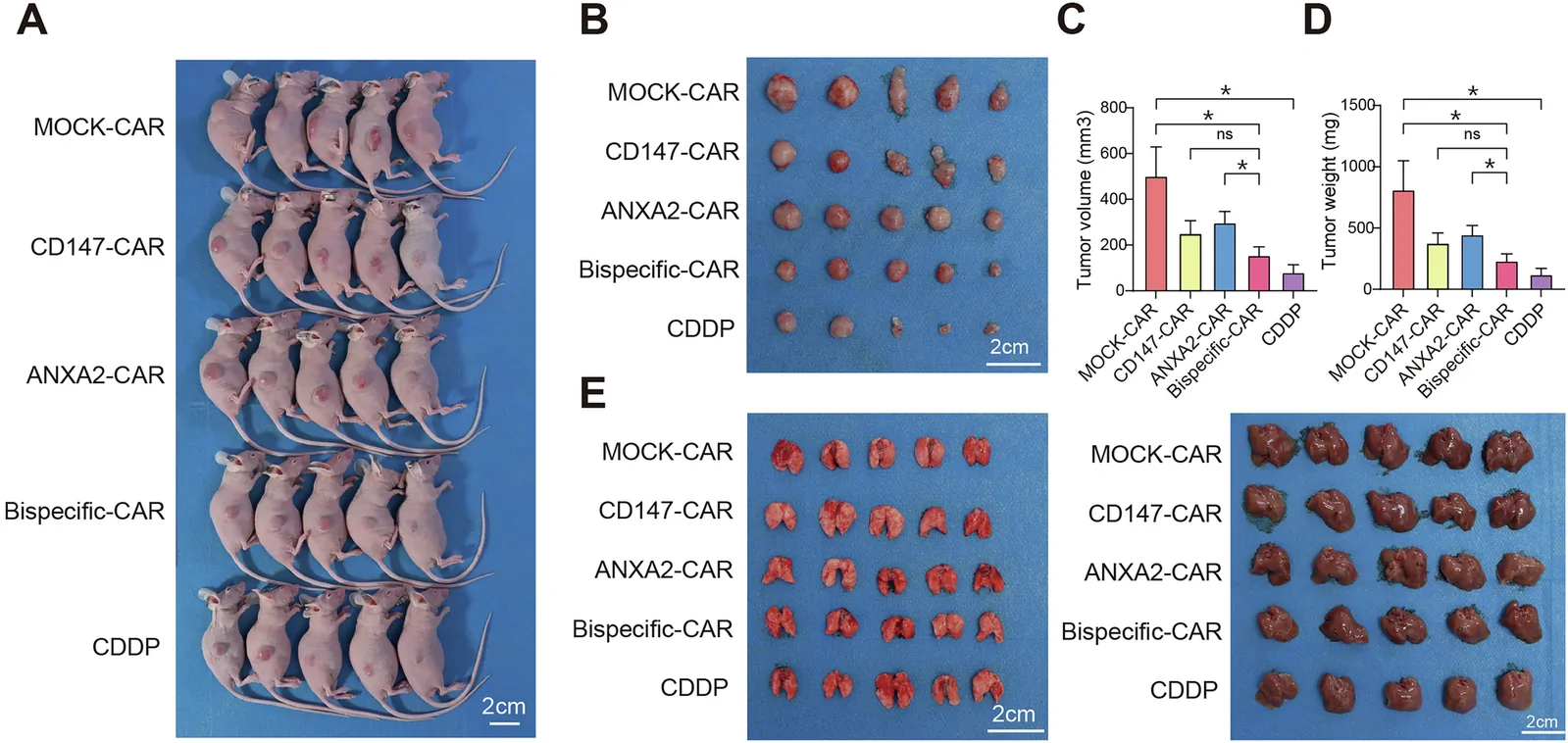
ANXA2-CD147-CAR-T cells suppress osteosarcoma growth in subcutaneous CDX models. **A**, **B** Representative images of tumor-bearing nude mice and excised tumors from MOCK-CAR-T, ANXA2-CAR-T, CD147-CAR-T, ANXA2-CD147-CAR-T, and cisplatin (CDDP, positive control) groups (*n* = 5). **C**, **D** Quantification of tumor volumes and weights. Tumors in the bispecific-CAR-T group were significantly smaller than those in ANXA2-CAR-T and MOCK groups. **E** Gross images of lung and liver tissues showing no obvious macroscopic toxicity in any group. *P < 0.05, ns not significant.

A subcutaneous cell-derived xenograft (CDX) model was established in nude mice to evaluate in vivo efficacy. Cisplatin treatment was included as a positive control. Tumor volume and weight in the bispecific-CAR-T group were significantly reduced compared with ANXA2-CAR-T and MOCK groups (Fig. 4A–D). Although the difference between bispecific-CAR-T and CD147-CAR-T did not reach statistical significance (*p* = 0.052), a trend favoring the bispecific construct was observed. Importantly, no apparent toxicity was detected in the lungs or liver (Fig. 4E). Collectively, these results demonstrate that bispecific CAR-T cells exert potent antitumor effects in vivo.

### Bispecific CAR-T cells inhibit osteosarcoma growth and lymph node metastasis

**Fig. 5:**
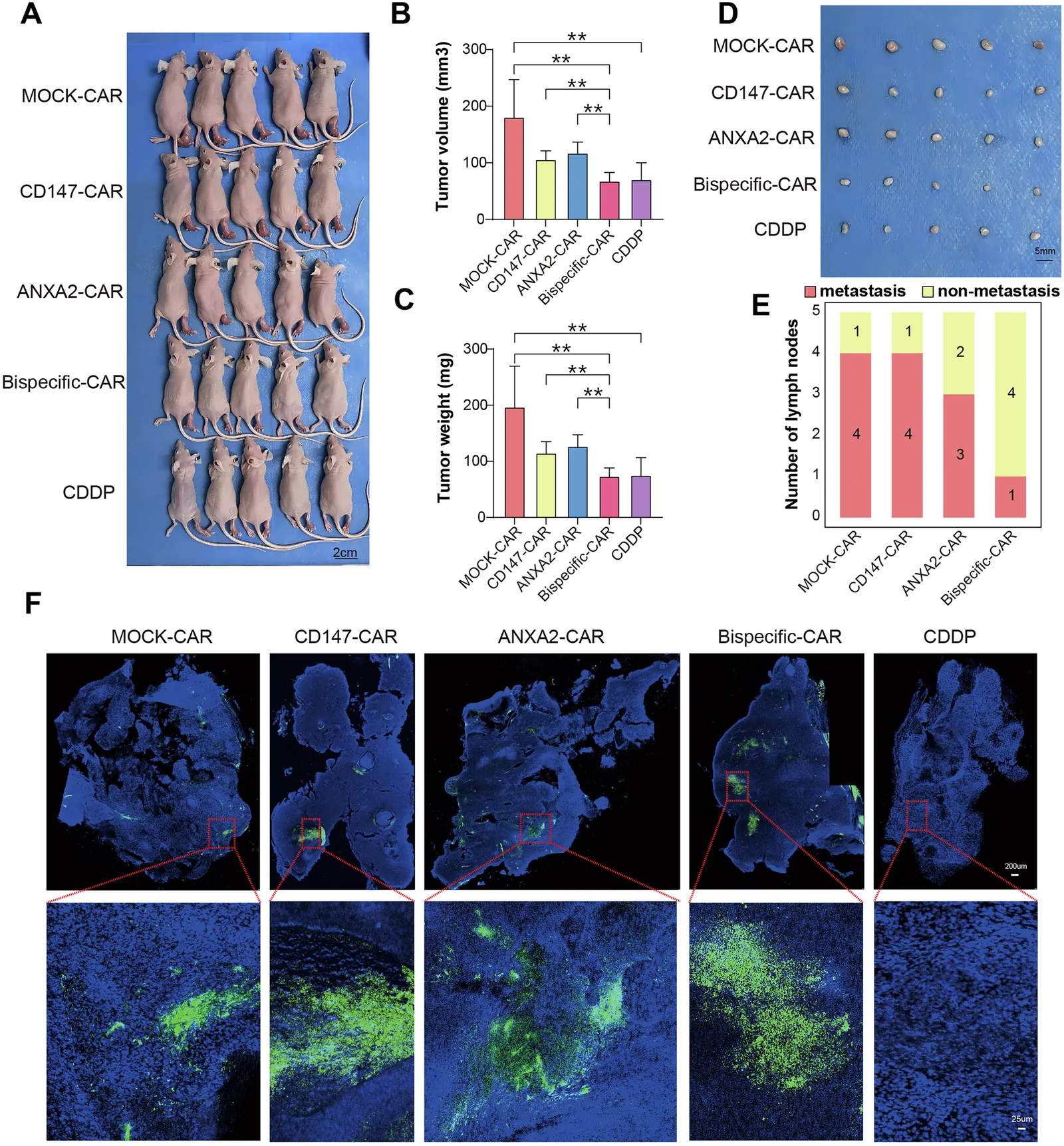
Bispecific CAR-T cells inhibit osteosarcoma growth and lymph node metastasis. **A** Representative images of tumor-bearing mice in the paw pad xenograft–inguinal lymph node metastasis model (*n* = 5). **B**, **C** Tumor volume and weight were significantly reduced in the bispecific-CAR-T group compared with monospecific and MOCK groups. **D** Representative images of excised inguinal lymph nodes. **E** Quantification of metastatic versus non-metastatic lymph nodes. **F** Confocal fluorescence microscopy showing GFP⁺ (green) CAR-T cells in tumor tissues. Bispecific-CAR-T–treated tumors exhibited markedly higher T-cell infiltration and expansion compared with other groups. Statistical analysis: Student’s *t* test was used for all comparisons. *P < 0.05, **P < 0.01, ***P < 0.001.

To further evaluate antimetastatic activity, a paw pad xenograft–inguinal lymph node metastasis model was established using 143B cells. Following CAR-T treatment, tumor volume and weight were significantly lower in the bispecific-CAR-T group compared with monospecific and MOCK groups (Fig. 5A–C). In addition, inguinal lymph nodes in bispecific-CAR-T–treated mice were markedly smaller than those in other groups (Fig. 5D).

H&E staining confirmed lymph node metastasis in only 1 of 5 bispecific-CAR-T–treated mice, compared with 3 in the ANXA2-CAR-T group and 4 each in the CD147-CAR-T and MOCK groups (Fig. 5E). Representative images of metastatic and non-metastatic lymph nodes are shown in Supplementary Fig. S4. Confocal microscopy revealed higher GFP fluorescence in tumors from bispecific-CAR-T–treated mice, indicating enhanced T-cell infiltration and expansion. These findings demonstrate that bispecific CAR-T cells not only inhibit primary tumor growth but also suppress lymph node metastasis.

### Bispecific CAR-T cells display strong antitumor activity in PDX models

**Fig. 6:**
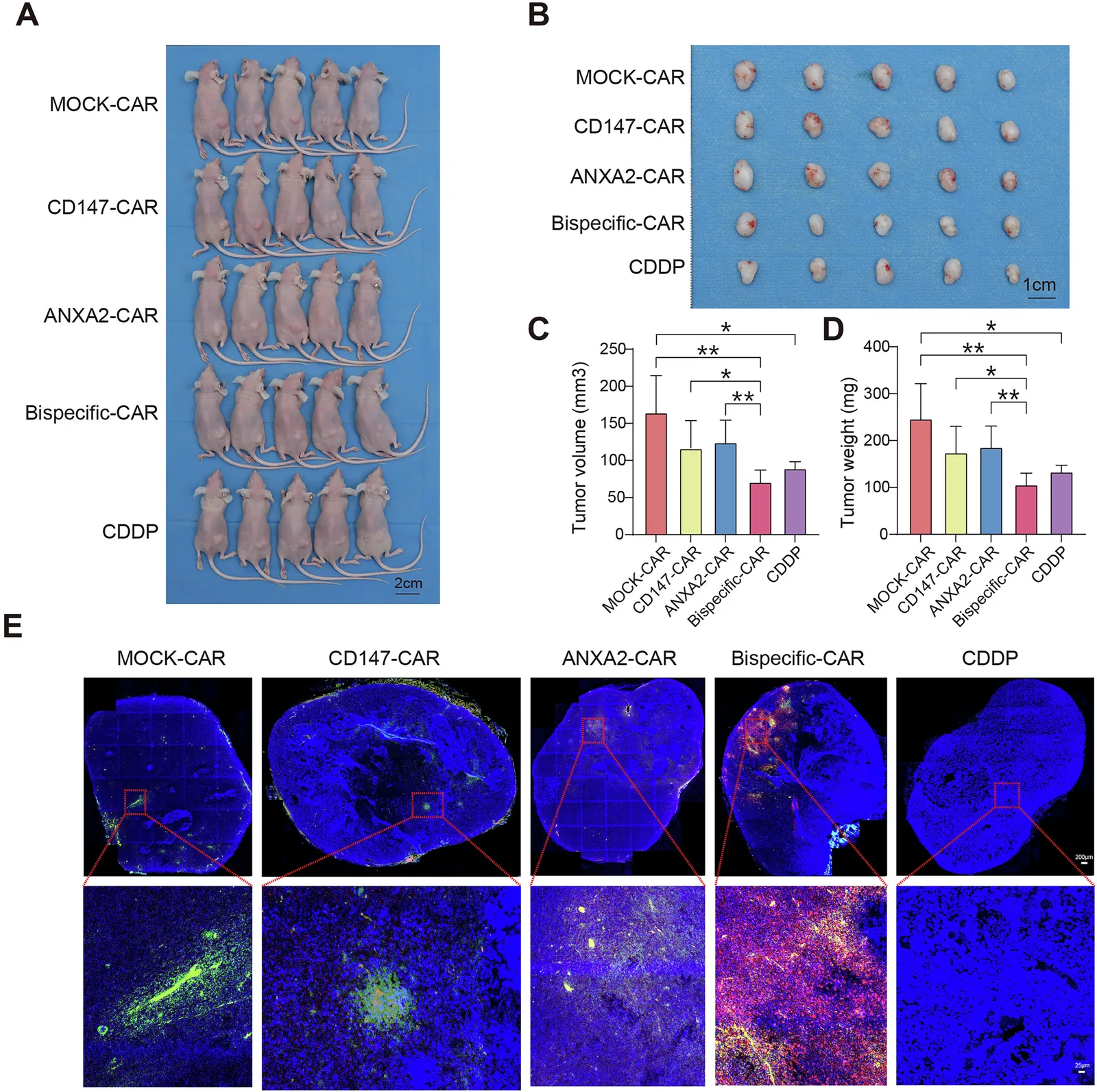
Bispecific CAR-T cells display strong antitumor activity in PDX models. **A**, **B** Representative images of tumor-bearing mice and excised tumors from patient-derived xenograft (PDX) models treated with different CAR-T cells or cisplatin (*n* = 5). **C**, **D** Tumor volumes and weights were significantly reduced in the bispecific-CAR-T group compared with monospecific and MOCK groups. **E** Confocal microscopy showing GFP (green) and CD3 (red) co-localization in tumors. The bispecific-CAR-T group exhibited the highest infiltration of GFP⁺CD3⁺ T cells. Statistical analysis: Student’s *t* test was used for all comparisons. *P < 0.05, **P < 0.01, ***P < 0.001.

Patient-derived xenograft (PDX) models were established by subcutaneous implantation of surgically resected osteosarcoma specimens into nude mice. PDX models provide clinically relevant predictive value and are considered essential for translational oncology research. In these models, bispecific-CAR-T treatment significantly reduced tumor volumes compared with monospecific and MOCK groups (Fig. 6A–D). Confocal imaging confirmed the highest levels of GFP⁺CD3⁺ T-cell infiltration in the bispecific-CAR-T group (Fig. 6E). In addition, liver tissues from mice in each group were collected and subjected to H&E staining, and no obvious liver injury was observed (Supplementary Fig. S5). These results further validate the potent in vivo antitumor efficacy of ANXA2-CD147 bispecific CAR-T cells.

### scRNA-seq and immunohistochemistry(IHC) reveals enhanced M1 macrophage infiltration in bispecific CAR-T–treated tumors

**Fig. 7:**
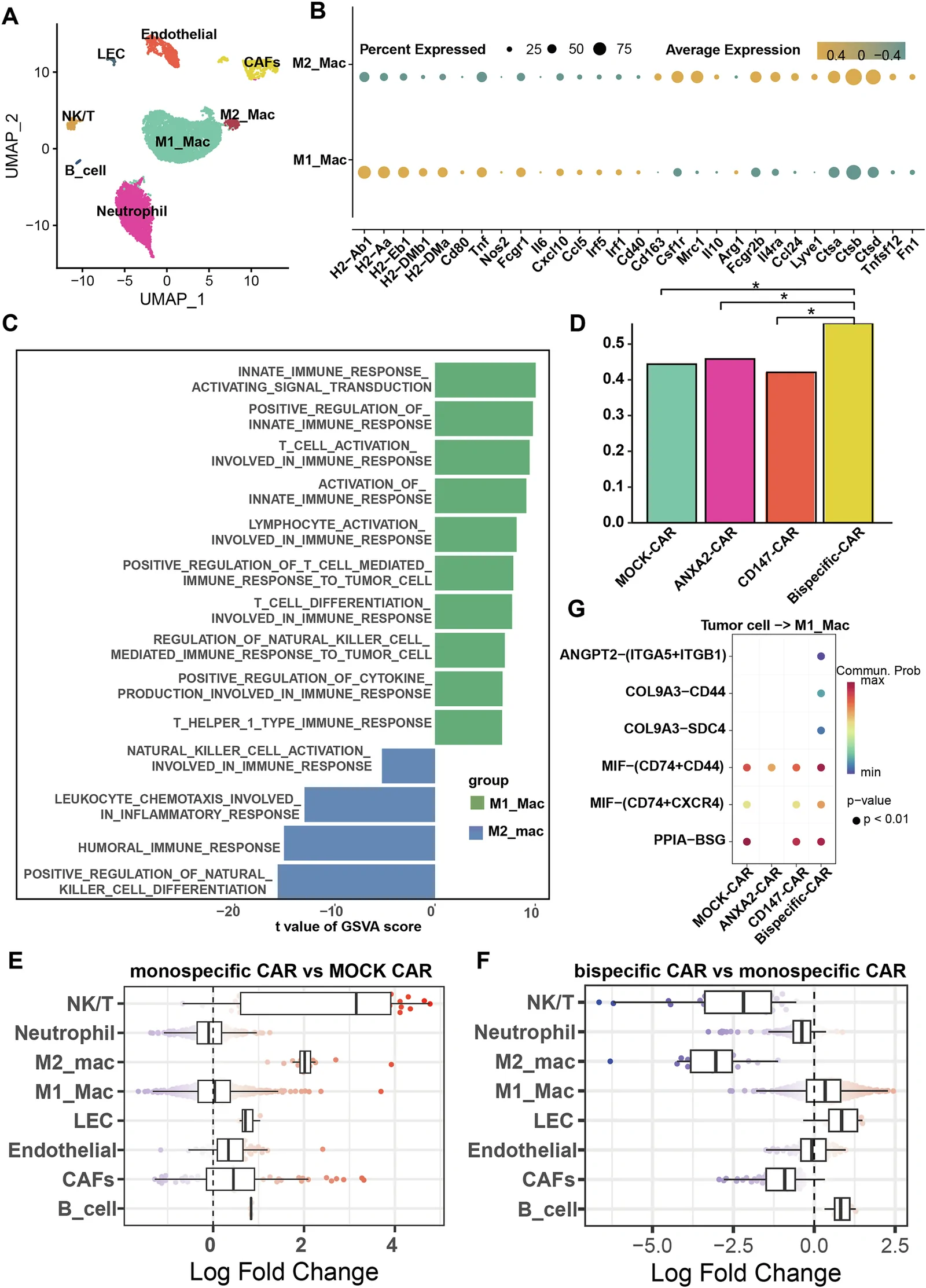
scRNA-seq reveals enhanced M1 macrophage infiltration in bispecific CAR-T–treated tumors. **A** UMAP visualization of scRNA-seq data from CDX tumors (*n* = 3) showing distinct immune cell clusters. **B** Dot plots of marker gene expression defining M1 macrophages (M1_Mac) and M2 macrophages (M2_Mac). **C** GSVA analysis demonstrating that M1_Mac were enriched in immune-activating pathways, including innate immune response, T-cell activation, and cytokine production. **D** Proportion of M1_Mac in each treatment group, showing significant increases in the bispecific-CAR-T group. **E**, **F** MiloR differential abundance analysis. Monospecific CAR-T increased M1_Mac infiltration compared with MOCK, while bispecific CAR-T induced a more pronounced enrichment. **G** Ligand–receptor analysis between M1_mac and tumor cell.

To investigate the effects of bispecific CAR-T cells on the tumor microenvironment, scRNA-seq was performed on tumors from MOCK (*n* = 3), ANXA2-CAR (*n* = 3), CD147-CAR (*n* = 3), and bispecific-CAR (*n* = 3) groups in CDX models. Mouse-derived cells were extracted from the cell_classification.csv output of CellRanger for tumor microenvironment analysis. After quality control, 10,158 cells were retained and classified into 8 clusters. Cluster C1 expressed H2-Ab1, H2-Aa, Cd74, Cd86, and Cd68, consistent with M1 macrophages (M1_Mac), whereas cluster C6 expressed Mrc1, Cd163, C1qa, and Lyve1, indicative of M2 macrophages (M2_Mac) (Fig. 7A, Supplementary Fig. S6A). Gene set scoring further confirmed C1 as M1 and C6 as M2 macrophages (Supplementary Fig. S6B).

M1_Mac cells exhibited high expression of H2-Ab1, H2-Aa, H2-Eb1, H2-DMb1, and H2-DMa, suggesting antigen-presenting capacity; Cd80, supporting T-cell activation; and Tnf, indicating direct cytotoxicity via TNFα/β secretion. Fcgr1 was also highly expressed, implying potential involvement in antibody-dependent cellular cytotoxicity (ADCC) (Fig. 7B). GSVA analysis revealed enrichment of immune-activating pathways, including innate immune response activating signal transduction, positive regulation of innate immune response, and T-cell activation involved in immune response (Fig. 7C).

Comparison of treatment groups showed that M1_Mac proportions were significantly increased in the bispecific-CAR-T group compared with monospecific and MOCK groups (Fig. 7D). Differential abundance analysis using MiloR further confirmed that while monospecific CAR-T increased M1_Mac infiltration relative to MOCK, bispecific-CAR-T treatment resulted in a more pronounced enrichment (Fig. 7E, F). Collectively, these findings suggest that bispecific CAR-T therapy enhances M1 macrophage infiltration, thereby promoting antitumor immunity. Ligand–receptor analysis indicated that bispecific-CAR-T cells strengthened interactions between tumor cells and M1 macrophages, including ANGPT2–(ITGA5 + ITGB1), COL9A3–CD44, and COL9A3–SDC4, suggesting that these interactions may contribute to enhanced M1 macrophage recruitment in the bispecific-CAR-T group (Fig. 7G).

**Fig. 8:**
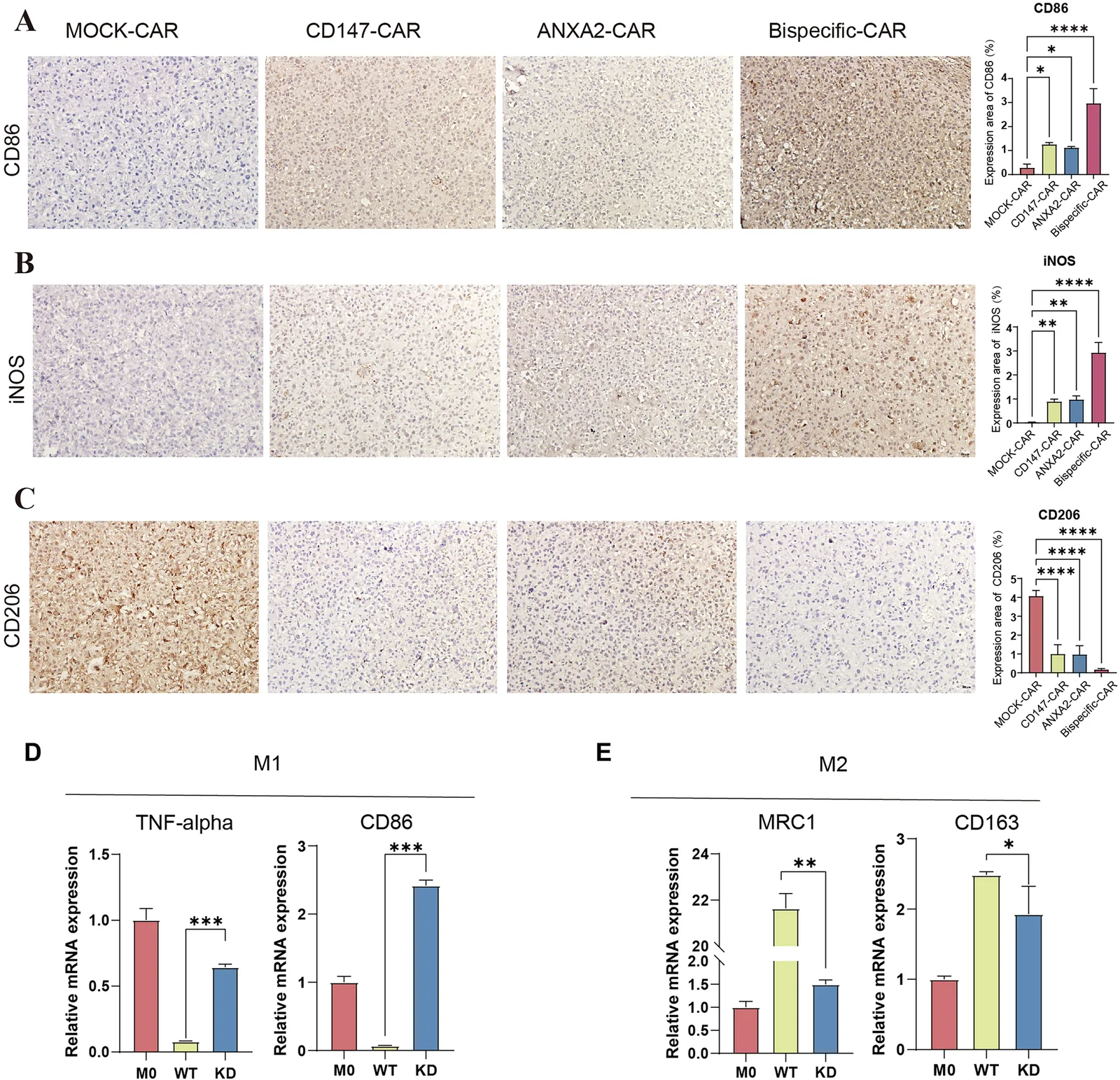
Experimental validation of macrophage polarization after bispecific CAR-T treatment. **A**–**C** Representative IHC images and quantification of CD86, iNOS, and CD206 expression in PDX tumor (*n* = 3) tissues from MOCK-CAR, CD147-CAR, ANXA2-CAR, and bispecific-CAR groups. CD86 and iNOS were increased, whereas CD206 was reduced, in the bispecific-CAR group. **D** qRT-PCR analysis of M1 macrophage marker genes TNF-alpha and CD86 in M0 macrophages co-cultured with wild-type 143B cells (WT) or CD147-knockdown 143B cells (KD) (*n* = 3). **E** qRT-PCR analysis of M2 macrophage marker genes MRC1 and CD163 in the same co-culture system (*n* = 3). Statistical analysis: Student’s *t* test was used for all comparisons. *P < 0.05, **P < 0.01, ***P < 0.001, ****P < 0.0001.

To further validate the single-cell RNA sequencing findings, immunohistochemistry was performed. Consistently, the expression of the M1 macrophage markers CD86 (Fig. 8A) and iNOS (Fig. 8B) was markedly elevated in the bispecific CAR-T group, while the expression of the M2 macrophage marker CD206 (Fig. 8C) was substantially reduced. These results further support the notion that bispecific CAR-T cells promote M1 macrophage infiltration. To verify the direct role of CD147 expressed by osteosarcoma cells in macrophage polarization, we used siRNA to silence CD147 expression in 143B cells. The conditioned media from CD147-silenced 143B cells (KD group) and wild-type 143B cells (WT group) were then used to treat macrophages, and the expression levels of M1/M2 polarization-related marker genes in macrophages were examined by qPCR. The results showed that the expression levels of the M1 macrophage markers TNF-α and CD86 were increased in the KD group compared with the WT group (Fig. 8D), whereas the expression levels of the M2 macrophage marker genes MRC1 and CD163 were downregulated in the KD group (Fig. 8E). These results suggest that osteosarcoma cells may suppress M1 polarization and promote M2 polarization through CD147, and that our CAR-T cells may promote M1 macrophage infiltration, at least in part, by targeting CD147.

## Discussion

In recent years, CAR-T therapy has emerged as a novel immunotherapeutic strategy, achieving remarkable success in hematological malignancies. In contrast, while preliminary therapeutic potential has been demonstrated in certain solid tumors, its broader application remains limited. For osteosarcoma, the highly complex tumor microenvironment and pronounced antigenic heterogeneity continue to pose substantial barriers to clinical translation. Thus, the development of more effective and safer CAR-T therapies is urgently needed [3, 22].

In this study, scRNA-seq was used to identify two tumor-associated antigens highly expressed in osteosarcoma cells. Based on these findings, a novel bispecific CAR-T construct—ANXA2/CD147 CAR-T—was generated and evaluated. Both in vitro and in multiple in vivo models, these engineered T cells demonstrated robust antitumor activity and inhibition of metastasis.

Because CAR-T cells rely on tumor antigens for recognition, the selection of suitable targets is critical for therapeutic efficacy. An optimal CAR-T target should meet several criteria: (i) broad coverage, with high expression across most tumor cells to ensure effective cytotoxicity [23]; (ii) strong specificity, being highly expressed in tumor tissues but absent or minimally expressed in normal tissues [24]; and (iii) stable and sustained expression, minimizing antigen escape. Annexin A2 (ANXA2), a 36-kDa member of the annexin family, satisfies many of these requirements. ANXA2 is frequently overexpressed in cancers such as esophageal [25], breast [26], and gastric cancer [27], where its upregulation correlates with poor prognosis. Functionally, ANXA2 promotes tumor proliferation, migration, and invasion [28, 29]. Prior studies have reported that ANXA2-CAR-T cells exert potent anti-ovarian cancer effects both in vitro and in vivo [20]. In the present work, ANXA2 was found to be highly expressed in osteosarcoma cells and was therefore selected as one of the CAR targets.

Cluster of differentiation 147 (CD147), also known as basigin (BSG), is a transmembrane glycoprotein belonging to the immunoglobulin superfamily. It is overexpressed in multiple malignancies and is closely associated with tumor initiation, progression, invasion, and metastasis, establishing it as an oncogene and a promising therapeutic target [30]. CD147 has already been applied as a CAR-T target in hepatocellular carcinoma, where anti-CD147 CAR-T cells displayed substantial antitumor efficacy [31]. However, its role in osteosarcoma has not previously been investigated. Our findings revealed high CD147 expression in both primary osteosarcoma tissues and metastatic lymph nodes, supporting its inclusion as the second target of this bispecific CAR strategy.

Solid tumors are characterized by profound heterogeneity, with distinct subpopulations exhibiting differential gene expression and antigen distribution [13, 14]. This diversity severely limits the efficacy of monospecific CAR-T therapies. Tandem CARs (TanCARs), which incorporate two scFvs within a single receptor in an “OR-gate” configuration, enable T-cell activation upon recognition of either antigen, thereby broadening target coverage and enhancing cytotoxicity. Previous studies have demonstrated that bispecific designs outperform single-target CAR-T in several solid tumors [15–17]. Yet, little is known about their application in osteosarcoma. In this study, ANXA2 and CD147 were combined in a novel tandem CAR design, significantly improving antigen recognition, tumor killing capacity, and suppression of metastasis.

Although osteosarcoma metastasis predominantly occurs through hematogenous dissemination, with the lung being the most common metastatic site, several studies have shown that regional lymph node metastasis can also occur in a subset of patients and is associated with extremely poor prognosis. Chinese investigators demonstrated, using both the SEER database and an independently collected multicenter cohort, that osteosarcoma patients with lymph node metastasis had markedly worse survival, with a 5-year survival rate of less than 20% [32]. In addition, Thampi et al. analyzed 2748 patients with high-grade osteosarcoma from the SEER database and found that regional lymph node involvement at diagnosis was rare, occurring in only 2.7% of patients; however, these patients had a 5-year overall survival rate of only 10.9%, compared with 54.3% in patients without nodal involvement, and regional lymph node involvement remained an independent adverse prognostic factor [33]. Therefore, investigating therapeutic strategies for lymph node metastasis in osteosarcoma is of considerable clinical significance. The bispecific CAR-T cells developed in this study were shown to suppress lymph node metastasis of osteosarcoma, possibly because both ANXA2 and CD147 are highly expressed in metastatic lymph nodes.

CAR-T cells can eliminate tumor cells through multiple mechanisms. First, upon antigen recognition, CAR-T cells become activated and release perforin and granzymes, thereby inducing tumor cell lysis [6]. Consistent with this mechanism, the bispecific ANXA2/CD147 CAR-T cells developed in this study secreted large amounts of GZMB, resulting in potent tumor cell killing. Second, CAR-T cells can promote tumor cell apoptosis by secreting cytokines such as IFN-γ and TNF-α [34, 35]. In this study, ANXA2/CD147 CAR-T cells secreted higher levels of TNF-α, thereby exerting antitumor effects. Moreover, the antitumor activity of CAR-T cells may also be associated with M1 macrophages. M1 macrophages activate T cells and assist in T-cell–mediated tumor killing, and are therefore generally considered tumor-suppressive [36]. Previous studies have shown that bispecific BCMA/CD24 CAR-T cells enhance M1 macrophage polarization [37]. Similarly, we found that ANXA2/CD147 CAR-T cells increased M1 macrophage infiltration. We propose that this increase may result from two mechanisms: on the one hand, TNF-α secreted by CAR-T cells can promote M1 macrophage polarization and infiltration; on the other hand, CD147 expressed on tumor cells may suppress M1 macrophage infiltration [38], while the CD147-targeting moiety of CAR-T cells may block this function, thereby increasing the abundance of M1 macrophages within tumors.

Nevertheless, several limitations should be acknowledged. First, although no toxicity was observed in vivo, systematic toxicological studies are required to confirm the safety profile of ANXA2/CD147-CAR-T cells. Second, the present work is preclinical, and clinical trials will be essential to establish therapeutic efficacy.

In conclusion, this study successfully developed a novel ANXA2/CD147 bispecific CAR-T cell therapy that demonstrated potent antitumor activity in vitro and in vivo. These findings provide a promising strategy for advancing immunotherapy in osteosarcoma.

## Supplementary information

Supplementary_Figure_legend

Figure S1

Figure S2

Figure S3

Figure S4

Figure S5

Figure S6

table_S1

table_S2

## Data Availability

The human scRNA-seq data have been deposited in Gene Expression Omnibus (GEO) under accession number GSE162454, and National Genomics Data Center (HRA007229 and HRA008101). We have also uploaded the mouse sequencing data to the Genome Sequence Archive (GSA), and the data are accessible at the following link: https://ngdc.cncb.ac.cn/gsa/browse/CRA030705.

## Code availability

All data analysis and processing were executed through the use of published software or packages, the details of which are referred to within Methods. No novel code or mathematical algorithms were generated by this study.
