# Supplementary material for: Bispecific ANXA2/CD147 CAR-T cell therapy for osteosarcoma
Source: Oncogene. 2026 Aug 3;45(38):4037–50. doi: 10.1038/s41388-026-03926-2 (PMC13577908; doi:10.1038/s41388-026-03926-2)
Supplement: Supplementary file 1 [file 41388_2026_3926_MOESM1_ESM.docx]

### ****Supplementary Figure S1. Expression of ANXA2 and CD147 in osteosarcoma versus femoral head tissues.****

**(A)** UMAP visualization of scRNA-seq data showing osteoblast and immune cell populations. **(B–C)** Violin plots showing significantly higher expression of ANXA2 and CD147 in osteosarcoma (OS) compared with femoral head (FH) controls.

### ****Supplementary Figure S2. Purity and morphology of transduced CAR-T cells.****

**(A)** Flow cytometry analysis showing CD3⁺ T-cell purity after magnetic bead sorting (93.4%). **(B)** Representative images of bispecific-CAR–transduced T cells.

### ****Supplementary Figure S3. Expression gradient of ANXA2 and CD147 in osteosarcoma cell lines.****

**(A)** Flow cytometry showing differential expression of ANXA2 and CD147 in 143B, SAOS2, and MG63 cells. **(B–C)** Quantification of mean fluorescence intensity (MFI).
**(D–E)** qRT-PCR validation of ANXA2 and CD147 expression, confirming the gradient pattern (143B > SAOS2 > MG63).

### ****Supplementary Figure S4. Histological validation of lymph node metastasis.****

**(A)** Representative H&E staining of metastatic and non-metastatic lymph nodes. Metastatic nodes exhibited atypical tumor cells with large, hyperchromatic nuclei, whereas non-metastatic nodes were predominantly lymphocytic without atypia.

**Supplementary Figure S5. Histological evaluation of liver toxicity in PDX model mice.**

Representative H&E staining images of liver tissues from MOCK-CAR, ANXA2-CAR, CD147-CAR, and ANXA2-CD147-CAR groups at 100x and 200x magnification. No obvious liver injury was observed in any group.

**Supplementary Figure S6. Additional scRNA-seq analyses of macrophage subclusters in CAR-T-treated tumors.**

(A) Dot plot showing the expression of canonical marker genes used to annotate cell clusters, including M1 macrophages and M2 macrophages. (B) Violin plots showing M1 and M2 macrophage signature scores in C1_M1_Mac and C6_M2_Mac clusters.
