# Supplementary material for: Bispecific ANXA2/CD147 CAR-T cell therapy for osteosarcoma
Source: Oncogene. 2026 Aug 3;45(38):4037–50. doi: 10.1038/s41388-026-03926-2 (PMC13577908; doi:10.1038/s41388-026-03926-2)
Supplement: Supplementary file 2 [file 41388_2026_3926_MOESM8_ESM.docx]

Table S1. Primer sequences used for qRT-PCR.

| Genes | Sequences |
| --- | --- |
| GAPDH | '5- CAGGAGGCATTGCTGATGAT -3' |
|  | '5- GAAGGCTGGGGCTCATTT -3' |
| ANXA2 | '5- TGGTCTCCCGCAGTGAAGTGG -3' |
|  | '5- GCTTTCTGGTAGTCGCCCTTAGTG -3' |
| CD147 | '5- CTGTTCGTGCTGCTGGGATTCG -3' |
|  | '5- TGGAGCCAAGGTCTTCTACGGTAG -3' |
| TNF-α | '5- CTTCCAGCTGGAGAAGGGTG -3' |
|  | '5- CCCAAAGTAGACCTGCCCAG -3' |
| IL6 | '5- AGTGAGGAACAAGCCAGAGC -3' |
|  | '5- GGTCAGGGGTGGTTATTGCA -3' |
| CD86 | '5- GGAAGAAGAAGAAGCGGCCT -3' |
|  | '5- CGCTGGGCTTCATCAGATCT -3' |
| MRC1 | '5- AGGATGGGTACTGGGCAGAT -3' |
|  | '5- CTGGACCTTGGCTTCGTGAT -3' |
| CD163 | '5- GCGGGAGAGTGGAAGTGAAA -3' |
|  | '5- ACCTGCACTGGAATTAGCCC -3' |
