# Supplementary material for: Bispecific ANXA2/CD147 CAR-T cell therapy for osteosarcoma
Source: Oncogene. 2026 Aug 3;45(38):4037–50. doi: 10.1038/s41388-026-03926-2 (PMC13577908; doi:10.1038/s41388-026-03926-2)
Supplement: Supplementary file 3 [file 41388_2026_3926_MOESM9_ESM.docx]

| Gene | Strand type | Sequence |
| --- | --- | --- |
| CD147-717 | Sense strand | GAAGUCGUCAGAACACAUCAATT |
|  | Antisense strand | UUGAUGUGUUCUGACGACUUCTT |
| CD147-460 | Sense strand | ACAGUCUUCACUACCGUAGAATT |
|  | Antisense strand | UUCUACGGUAGUGAAGACUGUTT |
| CD147-891 | Sense strand | GCUACACAUUGAGAACCUGAATT |
|  | Antisense strand | UUCAGGUUCUCAAUGUGUAGCTT |

Table S2. Sequences of siRNAs used for CD147 knockdown.
